# Editorial

**Published:** 1868-07-01

**Authors:** 


### *EDITORIAL.*

### *Items, News, and Gossip.*

THE Supreme Court of Michigan "reserves its decision" on the question whether the "vinegar factory" established at Grand Rapids by the Board of Regents of the University at Ann Arbor is or is not a *Department*, in the eye of the law, of the latter concern. It is *still* in abeyance. Several years ago it will be recollected the same court was "equally divided in its combined mind on the Prohibitory Liquor Law." The old question now comes up in a new shape.——The resignations of Profs. Armor and Greene have been accepted. Prof. Sager still doubts, and Prof. Ford is at present in very deep water. ——The best essay on Medical Education we have recently read we reproduce bodily from the *Medical Gazette*:——"Many of our exchanges discuss the question of medical education from various points of view. It seems to us that there are two indications to be fulfilled; first, to select students with enough brain-room to contain the necessary amount of knowledge, and second, to fill that brain-room with proper material. The difficulty lies rather with the learners than with the teachers in every profession. It is impossible to get a pint of cream into a gill measure."——To which, however, we add: It is as difficult to get cream from some *cattle* as "blood from a turnip."—— Liebig's artificial milk is an imitation of the natural milk, as near as chemistry can make it. Boil ½ oz. of wheat flour to a paste in 5 oz. of skimmed milk. Then add ½ oz. bruised malt, 1 oz. water, and a solution of 3 grammes (about 20 grains) of bicarb. pot. in 11 parts water. Keep this in a bottle surrounded with warm water till it gets creamy. Then put it on a fire, and then strain it through a fine sieve.——Prof. Robley Dunglison has resigned the position he has long and illustriously filled in Jefferson Medical College.——Clarke's process of preserving bodies, mainly by carbolic acid, proves very successful. At Bellevue an autopsy was made, and the tissues found perfectly fresh, although 107 days after death. We shall publish the process next number.

We trust some of our readers will give us the results of their observations on the pernicious effects of the too long continued and excessive use of *Bromide of Potassium.* We have seen those of very disastrous character, but our profound regard for the code of ethics temporarily obliges us to abstain from details. ——A little *Ether* in the bottle containing Ergot or similar substances, liable to destruction by insects, will be found a good preservative.——The *Pacific Medical and Surgical Journal* now reaches our sanctum regularly, and we take pleasure in saying, is one of our most valued and valuable exchanges. It is published at San Francisco by Bancroft & Co., and edited by the Drs. Gibbons. $5.00 a year, postage prepaid.

The Illinois State Dental Society held its fifth annual meeting at Springfield, May 12, 1868. At the last meeting of this Society, Drs. French and Smith were appointed to present subjects for discussion. The topics embraced Facial Neuralgia, Operative Dentistry, Treatment of Decayed Six Year Molars, Plugging Pulp Cavities and Canals, Receding of Gums, etc., Local and General Anæsthesia, Vulcanite, Gold Plates, and miscellaneous business. We have been informed that the diligent members of this Society had a well attended and instructive session. The subjects were reviewed fully, and much light shed upon the details of the science.

The New York *Medical Gazette* passes under the control of A. L. Carroll, M.D. His salutatory is brief and to the point:

The most notable event of the week, as regards this periodical, has been the shifting of its editorial responsibilities upon new shoulders; a transition effected with such volcanic rapidity that its strata may be found, perhaps, somewhat distorted. We therefore crave our readers' indulgence for any plutonic ruggedness of aspect in our literary landscape this week, and hope, hereafter, to smoothe its surface with fertile deposit.

We have authority for stating that Dr. Duffield's Vacuo-Maceration Process is not patented. A full description is given on page 275 of April 15th No. of Medical Journal.

### *North-western Medical Agency.*

The Journal takes pleasure in directing attention to the advertisement of Messrs. Walker & Son, on our advertising pages. The profession will find them thoroughly competent and reliable. Orders of all kinds may be entrusted to them with entire confidence that they will be attended with promptness, sound judgment, and at reasonable rates.

H. V. PASSAGE, M.D., of Peru, Ind., in a private note, says: "Mr. Stohlman, of the firm of Tieman & Co., N.Y., made the first hypodermic syringe at the request, and for the use, of the late Prof. Brainard, *over twenty years ago*. The French condemned the instrument when it was exhibited to them and its uses explained by Dr. B., but long afterwards claimed it as a specimen of French invention."

THE prospects of *Rush Medical College* were never more flattering than at the present. The applications for circulars and letters are multitudinous from every part of the country. The present Editor thinks this is because the profession recognize the fact that its Faculty attend to their business as such, and do not meddle with *isms* and Utopian schemes hatched in empty heads. The best kind of *reform* begins at home.

CORRESPONDENTS, we trust, we trust, will still bear with us. Their favors are duly appreciated, and will appear as space permits. LOOT will be afforded in abundance shortly.

